# Measurement of the Perception of Control during Continuous Movement using Electroencephalography

**DOI:** 10.3389/fnhum.2017.00392

**Published:** 2017-07-27

**Authors:** Wen Wen, Atsushi Yamashita, Hajime Asama

**Affiliations:** Department of Precision Engineering, University of Tokyo Tokyo, Japan

**Keywords:** sense of control, EEG, event-related potential, alpha-mu rhythm, attention

## Abstract

“Sense of control” refers to the subjective feeling of control over external events. Numerous neuropsychological studies have investigated the neural basis of the sense of control during action performance; however, most previous studies have focused on responses to a single discrete action outcome rather than real-time processing of action-outcome sequences. In the present study, we aimed to identify whether certain patterns of brain activation are associated with the perceived control during continuous movement. We recorded electroencephalography (EEG) signals while participants continuously moved a right-handed mouse in an attempt to control multiple visual stimuli. When participants perceived a sense of control over the stimuli, we observed a positive potential approximately 550 ms after the onset of movement, while no similar potential was observed when participants reported a lack of control. The appearance of this potential was consistent with the time window of awareness of control in a behavioral test using the same task, and likely reflected the explicit allocation of attention to control. Moreover, we found that the alpha-mu rhythm, which is linked to sensorimotor processing, was significantly suppressed after participants came to a conclusion regarding the level of control, regardless of whether control or lack of control was perceived. In summary, our results suggest that the late positive potential after the onset of the movement and the suppression of alpha-mu rhythm can be used as markers of the perception of control during continuous action performance and feedback monitoring.

## Introduction

“Sense of control” refers to the subjective feeling of being in control of one’s own actions and their effects on external events. In psychosocial terms, a sense of control is important for understanding and interpreting changes in the external environment, and for behavioral adaptation in response to such changes. Generally, a sense of control emerges from the appropriate linkage of motor command-based predictions and actual sensory feedback, whereas a delusion of control arises from a failure to internally represent the predicted consequence of an action (i.e., the comparator model, Frith et al., 2000a,b).

Previous studies have investigated the brain regions and activation patterns associated with the sense of control. For instance, with respect to the comparator model, the right posterior parietal cortex (Fink et al., 1999; Farrer and Frith, 2002; Blakemore and Sirigu, 2003; Farrer et al., 2003, 2008; Yomogida et al., 2010) and pre-supplementary motor area (Moore et al., 2010; Tsakiris et al., 2010; Yomogida et al., 2010) have been associated with discrepancies between intended movements and actual sensory feedback. Further, electroencephalography (EEG) studies have indicated that the N1 component—a negative potential occurring approximately 100 ms after stimulus onset—is attenuated during self-produced or predicted events, relative to that observed during externally generated feedback (Kühn et al., 2011; Gentsch et al., 2012; Timm et al., 2014). Moreover, the sense of control has been associated with enhancements in feedback correct-related positivity (fCRP, a positive potential occurring approximately 225 ms post-stimulus) and attenuations in P3a (a positive potential occurring approximately 350 ms post-stimulus; Kühn et al., 2011; Bednark and Franz, 2014). Notably, most of these previous studies compared brain activity in response to sensory feedback between conditions that did or did not produce a sense of control. However, no studies to date have examined such differences in the experience of control during continuous action performance.

In everyday life, control over novel external objects is usually determined based on feedback received in response to repeated action performance. Previous studies have suggested that perceptual motor control is triggered by goal-level control, and that the sense of agency (i.e., the feeling of being the agent of control) exhibits a hierarchical relationship with the perceived level of control (Kumar and Srinivasan, 2014, 2017). Additional research has suggested that this sense of agency is dependent upon real-time monitoring of the level of control (Caspar et al., 2016). In the present study, we aimed to identify whether certain patterns of brain activation are associated with the level of perceived control, and to determine whether activity associated with successful matching between sensory feedback and actions can be used as a marker of the sense of control. To achieve the aforementioned aims, we designed a task in which participants continuously moved a mouse to determine whether or not they had control over several dots on a computer screen. We compared EEG signals during the mouse movement between conditions during which control was and was not experienced.

One previous study suggested that the sense of control enhances self-recognition by attracting attention, in what is known as the “self pop-out” effect (Salomon et al., 2013). Therefore, we predicted that attention related potentials, such as P300, might be triggered by the perception of control during movement. We calculated event-related potentials (ERPs) after the onset of movement within the time windows in which the sense of control probably emerged. In another previous study, Kang et al. (2015) combined EEG measurement with virtual reality, observing a decrease in alpha band power when participants had higher levels of control over a virtual hand. This finding indicate that alpha-mu suppression (Chatrian et al., 1959), may be associated with the sense of control. Therefore, we measured the alpha-mu rhythm at the C3 electrode and compared it between control conditions, as previous studies have reported that alpha-mu suppression originates in the left hemisphere (Bai et al., 2005) and can be detected at the C3 electrode during right hand movement (Oberman et al., 2005; Ulloa and Pineda, 2007; Woodruff et al., 2011).

## Materials and Methods

### Participants

The present study included two tasks: an EEG measurement task and a behavioral task. Thirteen right-handed students with normal or corrected-to-normal vision participated in the EEG measurement task (mean age = 23.4 ± 2.1 years), nine of whom also participated in the behavioral experiment (mean age = 24.1 ± 2.0 years). As we did not examine mouse movement during EEG measurement, 10 additional participants (mean age = 22.3 ± 2.1 years) performed the EEG task without undergoing EEG measurement. The study was approved by the ethics committee of the School of Engineering at the University of Tokyo. Written informed consent was obtained from all participants prior to study participation.

### Task and Procedure

**Figure 1:**
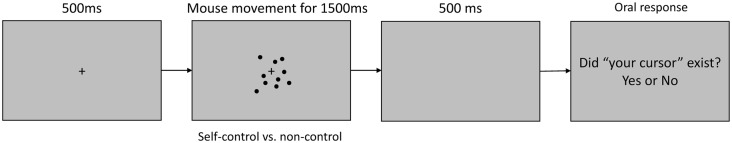
Timeline of a trial in the electroencephalography (EEG) task. At the beginning of each trial, a blank screen was presented for 500 ms, following which a central fixation cross and 12 surrounding black dots were displayed. The dots moved in correspondence with the onset and offset of mouse movement. In the self-control condition, one dot moved in correspondence with the direction and speed of mouse movement, while the other dots moved in random directions. In the non-control condition, all dots moved in random directions, independently of the direction or speed of mouse movement. Participants moved the mouse freely for 1500 ms, following which they were asked to orally report their sense of control over the dots with a “yes” or “no” response.

Visual stimuli were displayed on a 597 mm × 336 mm (1920 × 1080 pixels) LCD screen, which was positioned approximately 50 cm from the participant’s head. Head movement and viewing distance were not restricted. The stimuli were presented on a 32-bit Windows workstation using MATLAB (2016a; The Mathworks Inc., Natick, MA, USA) equipped with Psychtoolbox. For each trial (Figure 1), a blank gray screen was presented for 500 ms, following which a gray background containing a fixation cross and 12 black dots (3-mm) was presented for 1500 ms. The dots appeared at random positions within a 4° visual angle and remained static until the participant moved a mouse with his or her right hand. In half of the trials (the self-control condition), one of the dots moved in correspondence with the direction and speed of the mouse movement, while the other dots moved at the same speed but in random directions. The computer-generated random movements changed direction frequently and thus appeared very different from movements initiated by participants. In the remaining trials (the non-control condition), all dots moved in random directions when the mouse was moved, such that participants were not granted a sense of control. Dots disappeared if they moved beyond the (approximate) 4° visual angle and appeared again if they re-entered the 4° visual angle.

For each behavioral session, participants were instructed to gaze at the fixation cross and to pay attention to all of the moving dots using their peripheral vision, avoiding eye movements or blinking during the trial. Participants were instructed to begin moving the mouse immediately after the dots appeared on the screen, and to click the mouse as soon as they realized whether or not they were able to control the movement of any of the dots. Participants were instructed to move the mouse freely, but to avoid large unidirectional movements that might move the dot outside of the visible area (within a 4° visual angle). Participants were also encouraged to make smooth movements such as circles rather than small shaking motions to avoid sudden changes in the visual stimuli. Participants were allowed to familiarize themselves with the motions of the mouse at liberty prior to practice sessions to ensure their ability to easily to determine whether they had control over the dots (no response was required). The dots disappeared from the screen 500 ms after clicking, and participants orally reported their experience of control over the dots with a “yes” or “no” response at the end of each trial. The task itself contained 80 trials (40 trials in each condition). Participants completed 20 practice trials before the task. No feedback was provided during the practice or actual trials, and the order in which the trials were presented was randomized.

EEG measurement trials utilized the same stimuli and instructions regarding mouse movement as the behavioral task, except that participants were not instructed to click the mouse at any point. In each trial, the fixation cross and dots disappeared 1500 ms after stimulus onset. Thus, participants only reported their experience of control over the dots with an oral “yes” or “no” response. The EEG task also contained 80 trials (40 trials in each condition).

Each EEG session was conducted for a single participant at a time in a quiet, electrically sheltered room. After being fitted with the EEG device and receiving an explanation of the task, participants completed 20 practice trials. Before each trial, the experimenter orally reminded participants that the trial was about to start and observed the electrooculography (EOG) data. When a steady level of EOG activity was obtained, the experimenter started the trial. After each trial, the experimenter recorded the participant’s oral responses regarding his or her sense of control. Participants were encouraged to blink their eyes between trials, and were given short breaks after every 10–20 trials or in response to unsteady EOG data to prevent eye fatigue. The EEG task was performed before the behavioral task for those individuals participating in both tasks.

### EEG Recording and Analysis

EEG data were obtained at a sampling rate of 512 Hz using an active EEG electrode system g.GAMMAsys (g.tec Medical Engineering GmbH, Austria) fitted onto a cap that was individually sized for each participant. MATLAB Simulink (R2014a) equipped with the Signal Processing Toolbox and DSP System Toolbox was used for recording. Electrodes were placed at Fp1, Fp2, F7, F3, F4, F8, FC5, FC1, FCz, FC2, FC6, T7, C3, C1, Cz, C2, C4, T8, CP5, CP1, CPz, CP2, CP6, P7, P3, Pz, P4, P8, O1 and O2 in accordance with the extended 10–20 system. Vertical and horizontal EOG data were recorded via electrodes attached below and lateral to the outer canthi of the right eye. All EEG recording electrodes were injected with conductive electrode gel, and electrode impedances were transformed to output impedances of 1 kΩ by the active electrodes. The ground and reference electrodes were placed at Fz and the right earlobe, respectively. An online band filter of 0.1–100 Hz and a notch filter of 50 Hz were applied during EEG recording.

Trials with incorrect agency responses or EOG voltage variations exceeding ±40 μV were excluded from the ERP analysis. Because each trial lasted only 1500 ms, participants were typically able to avoid eye blinking and eye movements in most trials. As a result, only 6.15% of trials were excluded from analysis. Movement onset time-locked ERPs were calculated using MATLAB (2016a). A low-pass Butterworth filter of 30 Hz and a 200-ms pre-movement baseline correction were applied offline.

Trials with EOG voltages exceeding ±40 μV were excluded from the time-frequency analysis, and no further filter was applied to the data. The time-frequency analysis was conducted via a Morlet wavelet time-frequency transformation using the MATLAB wavelet toolbox (R2016a). We used wavelets with a 6-cycle width and frequencies ranging from 1 Hz to 60 Hz with varied scales (with a better scale resolution but poorer frequency resolution for lower frequencies, and vice versa for higher frequencies). The wavelet transform was applied to continuous signals from all trials for each participant to avoid the edge effect (i.e., distortion near the start and end of a given time-window). Power was defined as the square of the absolute value of the wavelet coefficient, and was normalized by dividing changes from baseline (50–250 ms prior to each trial) by the standard deviation of power within each trial period. Negative values indicated suppression from baseline.

## Results

### Behavioral Results

We conducted behavioral sessions to examine the time course of the experience of control and identify a time-window of focus for the EEG task. Mean latency to click responses was 763 ± 117 ms and 1036 ± 223 ms after the initiation of mouse movement in the self-control and non-control conditions (incorrect trials excluded), respectively. The difference between control conditions was significant (*t*_(8)_ = 4.65, *p* = 0.002, Cohen’*d* = 1.55). According to Libet’s theory regarding the temporal features of the intention to act vs. the initiation of voluntary action, the interval between awareness of a desire to act and actual action is approximately 200 ms (Libet, 1999). Thus, our findings suggest that participants came to a conclusion regarding their level of control 560 and 830 ms after the initiation of mouse movement in the self-control and non-control conditions, respectively. We used these time windows to examine whether brain activity changed along with the awareness of control. However, since we were unable to control for or measure individual differences and inter-trial variability, these time windows do not provide precise temporal information regarding the experience of control, but rather provide a rough division between pre- and post-sense-of-control states. In addition, response accuracy regarding control was 99.6% ± 0.6% and 99.2% ± 1.4% in the behavioral and EEG tasks, respectively.

### ERPs

**Figure 2:**
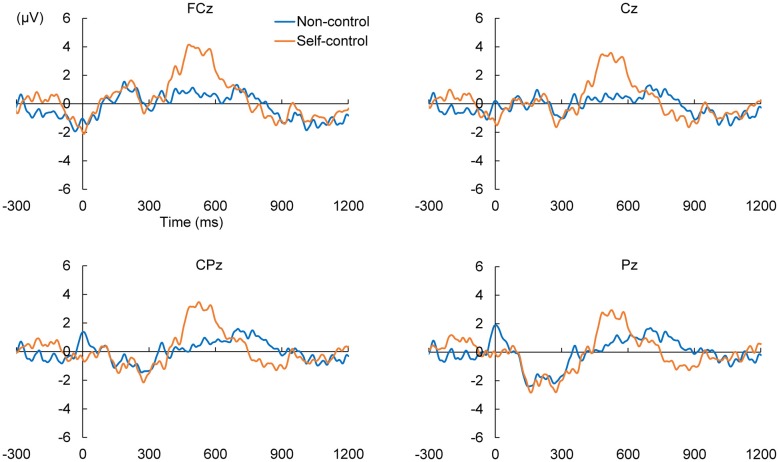
Grand average event-related potentials (ERPs) in self-control and non-control trials from 300 ms before the onset of mouse movement to 1200 ms after onset for electrodes along the midline.

**Figure 3:**
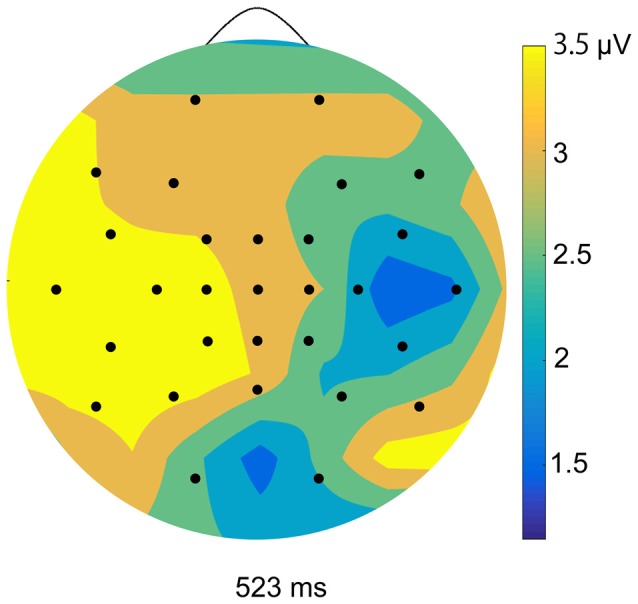
Topographical maps of peak amplitudes for the P500 component in the self-control condition.

We calculated ERPs after movement onset to verify the critical aspects of perceptual and attentional processes during the generation of sense of control. Trials with incorrect responses regarding the level of control were excluded. Figure 2 shows the grand average ERPs of electrodes along the midline (FCz, Cz, CPz and Pz) to the onset of movement in the two conditions of the EEG task. An obvious positive potential with a peak around 550 ms after the onset of movement was observed in the self-control condition but not in the non-control condition. This potential was designated “P500”. The scalp topography of the P500 potential is illustrated in Figure 3. A 2 × 4 (control × electrode) repeated measures analysis of variance (ANOVA) on peak amplitude values of P500 (450–650 ms after the onset of movement) at FCz, Cz, CPz and Pz revealed a significant main effect of control (*F*_(1,12)_ = 13.14, *p* = 0.003, $\eta_{\text{p}}^{2}$ = 0.523), while there was no main effect of electrode or interaction between control response and electrode (*F*_(3,36)_ = 0.84, *p* = 0.479, $\eta_{\text{p}}^{2}$ = 0.066; *F*_(3,36)_ = 2.05, *p* = 0.124, $\eta_{\text{p}}^{2}$ = 0.146, respectively). Higher P500 amplitudes were observed in the self-control condition than in the non-control condition. Importantly, the peak latency of P500 was consistent with the approximate time window during which the sense of control developed, suggesting that the P500 potential is associated with control-triggered attention.

We also examined whether lack of perceived control was associated with a specific potential within the corresponding time window. Therefore, we compared the positive and negative peaks 630–1130 ms after the onset of movement between the self-control and non-control conditions by conducting the same 2 × 4 repeated-measures ANOVAs as previously mentioned. No main effect of control response was observed for either the positive (*F*_(1,12)_ = 0.027, *p* = 0.871, $\eta_{\text{p}}^{2}$ = 0.002) or negative peak in this time window (*F*_(1,12)_ = 1.95, *p* = 0.188, $\eta_{\text{p}}^{2}$ = 0.140), suggesting that lack of perceived control was not associated with any attention-related potentials.

### Time-Frequency Analysis

**Figure 4:**
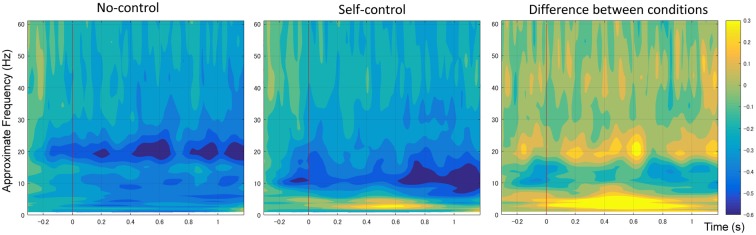
Average spectral power of 1–60 Hz frequency bands at the C3 electrode time-locked to movement onset in non-control and self-control trials. The contrast between the conditions is also shown.

Figure 4 shows the mean time-frequency power to the onset of movement in the non-control and self-control conditions at the C3 electrode. We first compared alpha-mu activity between the self-control and non-control conditions for the whole trial. Our findings indicated that suppression of alpha-mu activity was stronger in the self-control condition than in the non-control condition (*t*_(12)_ = 2.42, *p* = 0.033, Cohen’s *d* = 0.670). Further, in order to examine whether alpha-mu activity is associated with the experience of control or non-control, we used the approximate time windows of pre-control/non-control and post-control/non-control (i.e., before and after 560 and 830 ms of movement for the self-control and non-control conditions, respectively). A 2 × 2 (control × period) repeated measures ANOVA revealed a significant main effect of period (*F*_(1,12)_ = 15.125, *p* = 0.002, $\eta_{\text{p}}^{2}$ = 0.558). Surprisingly, however, no significant main effect of control or interaction between control and period was observed (*F*_(1,12)_ = 2.77, *p* = 0.112, $\eta_{\text{p}}^{2}$ = 0.187; *F*_(1,12)_ = 0.407, *p* = 0.536, $\eta_{\text{p}}^{2}$ = 0.033, respectively). These findings suggest that suppression of alpha-mu activity was stronger when participants became aware of their level of control, regardless of whether they perceived that they had or did not have control over the stimuli.

We conducted an additional behavioral task involving 10 new participants to examine whether mouse movement differed between self-control and non-control conditions (as we did not do so during the original EEG task). Our results indicated that participants moved the mouse less in the self-control condition than in the non-control condition (*t*_(9)_ = 4.33, *p* = 0.002). Such findings suggest that participants moved the mouse with greater intention to confirm their lack of control once they had achieved awareness.

Additionally, an increase in the power of the delta/theta band (1–7 Hz) was observed in the self-control condition relative to the non-control condition at the central, central-parietal and parietal regions (including C3, C1, C1, Cz, C4, CP5, CP1, CPz, CP2, CP6, P7, P3, Pz, P4 and P8). As previous research has indicated that increases in lower frequency band power are likely associated with differences in ERPs between conditions (Roach and Mathalon, 2008), we do not discuss these results further.

## Discussion

In the present study, we utilized EEG to examine the perception of control during continuous movement. We designed an experimental task in which participants continuously moved a mouse with their right hand in order to determine whether they had control over the stimuli. In the behavioral experiment, the average response time to determine the level of control was 763 and 1036 ms in the self-control and non-control conditions, respectively. These findings suggest that, after accounting for the temporal features of the intention to act in relation to the initiation of action, participants made conclusions regarding the level of control at approximately 560 and 830 ms after the onset of continuous movement in the self-control and non-control conditions, respectively. A positive ERP with a peak amplitude appearing about 550 ms after the onset of mouse movement was observed in the self-control condition. This ERP was likely associated with control-triggered attention. Moreover, time-frequency analysis revealed that, although alpha-mu activity was lower overall for self-control trials than non-control trials, alpha-mu activity decreased significantly after the participants became aware of their level of control in both conditions.

The present study was also the first to examine ERPs associated with the sense of control during continuous movement. Previous ERP studies have reported that self-generated or self-contributed events result in the attenuation of the N1 and P3 potentials (Kühn et al., 2011; Gentsch et al., 2012; Timm et al., 2014). These two potentials are associated with unexpected outcomes and have thus been linked to agency errors. In contrast, we focused on components associated with the awareness of control during continuous movement rather than the sensory processing of a single discrete action outcome. Our findings of P500 suggest that attention and attention-related brain activity can be used as markers of the sense of control, as no such activity was observed when participants perceived a lack of control. Further, our findings indicated that people likely pay greater attention on an object that is under control than uncontrollable objects. However, further studies are required to elucidate the neural processes underlying the association between attention and sense of control.

Furthermore, our findings demonstrated that alpha-mu suppression/desynchronization during continuous movement was associated with the experience of control, regardless of whether participants actually had control over the movement. Alpha-mu event-related desynchronization can be induced by motor preparation (Leocani et al., 1997; Cochin et al., 1999; Ramoser et al., 2000; Muthukumaraswamy et al., 2004; Pineda, 2005) and selective attention (Van Winsun et al., 1984; Dujardin et al., 1993; Suffczynski et al., 2001; Polich, 2007). In general, desynchronization begins 2 s before movement onset over the contralateral sensorimotor cortex (Pineda, 2005), and the magnitude of alpha-mu rhythm desynchronization reflects task complexity as well as the brain areas involved (Boiten et al., 1992; Dujardin et al., 1995). Our additional behavioral results indicated that participants moved the mouse less during the self-control condition than during the non-control condition, indicating that they engaged in more careful motor planning after becoming aware of control. Thus, stronger alpha-mu suppression may be the result of more accurate and intentional motion planning after people came to a conclusion regarding the level of control.

In summary, the present study is the first to examine the neural mechanisms underlying the experience of control during continuous movement. Our data highlight two EEG features associated with the perception of control: a positive potential appearing approximately 550 ms after the onset of movement, which is presumed to reflect increases in attention triggered by the awareness of control, and enhancements in alpha-mu rhythm desynchronization, which were associated with awareness of both control and lack of control. We believe that our findings are important for understanding the neural basis of the sense of control may be useful for decoding neural signals associated with the perception of control in future machine learning studies.

## Author Contributions

WW, AY and HA designed this work; wrote the manuscript. WW performed the experiment and analyses.

## Conflict of Interest Statement

The authors declare that the research was conducted in the absence of any commercial or financial relationships that could be construed as a potential conflict of interest.
